# Monoclonal Antibody: A New Treatment Strategy against Multiple Myeloma

**DOI:** 10.3390/antib6040018

**Published:** 2017-11-14

**Authors:** Shih-Feng Cho, Liang Lin, Lijie Xing, Tengteng Yu, Kenneth Wen, Kenneth C. Anderson, Yu-Tzu Tai

**Affiliations:** 1 Division of Hematology & Oncology, Department of Internal Medicine, Kaohsiung Medical University Hospital, Kaohsiung Medical University, Kaohsiung 807, Taiwan; shih-feng_cho@dfci.harvard.edu; 2 Faculty of Medicine, College of Medicine, Kaohsiung Medical University, Kaohsiung 807, Taiwan; 3 LeBow Institute for Myeloma Therapeutics and Jerome Lipper Multiple Myeloma Center, Dana-Farber Cancer Institute, Harvard Medical School, Boston, MA 02215, USA; liang_lin@dfci.harvard.edu (L.L.); lijie_xing@dfci.harvard.edu (L.X.); tengteng_yu@dfci.harvard.edu (T.Y.); kenneth_wen@dfci.harvard.edu (K.W.); Kenneth_anderson@dfci.harvard.edu (K.C.A.); 4 Department of Hematology, Shandong Provincial Hospital Affiliated to Shandong University, No. 324, Jingwu Road, Jinan 250021, China

**Keywords:** multiple myeloma, monoclonal antibody, immunomodulatory activity, bone marrow microenvironment

## Abstract

2015 was a groundbreaking year for the multiple myeloma community partly due to the breakthrough approval of the first two monoclonal antibodies in the treatment for patients with relapsed and refractory disease. Despite early disappointments, monoclonal antibodies targeting CD38 (daratumumab) and signaling lymphocytic activation molecule F7 (SLAMF7) (elotuzumab) have become available for patients with multiple myeloma in the same year. Specifically, phase 3 clinical trials of combination therapies incorporating daratumumab or elotuzumab indicate both efficacy and a very favorable toxicity profile. These therapeutic monoclonal antibodies for multiple myeloma can kill target cells via antibody-dependent cell-mediated cytotoxicity, complement-dependent cytotoxicity, and antibody-dependent phagocytosis, as well as by direct blockade of signaling cascades. In addition, their immunomodulatory effects may simultaneously inhibit the immunosuppressive bone marrow microenvironment and restore the key function of immune effector cells. In this review, we focus on monoclonal antibodies that have shown clinical efficacy or promising preclinical anti-multiple myeloma activities that warrant further clinical development. We summarize mechanisms that account for the in vitro and in vivo anti-myeloma effects of these monoclonal antibodies, as well as relevant preclinical and clinical results. Monoclonal antibody-based immunotherapies have already and will continue to transform the treatment landscape in multiple myeloma.

## 1. Introduction

Multiple myeloma is the second most common hematologic malignancy, characterized by the proliferation of malignant plasma cells in the bone marrow and excessive production of immunoglobulins [1,2]. The clinical outcome of patients with multiple myeloma has been improved in recent decades due to the development of novel therapeutic agents such as the proteasome inhibitors bortezomib [3], carfilzomib [4,5], and ixazomib [6] or immunomodulatory drugs (IMiDs) including thalidomide [7], lenalidomide [8], and pomalidomide [9,10]. With the incorporation of these novel agents into myeloma treatment strategies, the response rate and extent, progression-free survival, and overall survival have also been significantly improved in newly diagnosed patients [11,12,13,14,15,16,17]. However, in most cases, it remains a chronic and incurable disease due to its typical pattern of remission and relapse [18,19]. In addition, patients with refractory disease or who relapse after treatment with proteasome inhibitors and IMiDs have a very poor prognosis [18,20]. Thus, exploring novel approaches targeting different mechanisms to overcome drug resistance and minimize disease relapse are urgently needed.

With increased understanding of the biology of the disease, the development and evolution of multiple myeloma has been closely linked to specific immune system impairments. Malignant plasma cells express lower levels of tumor antigens and human leukocyte antigen (HLA) molecules [21,22], as well as higher levels of programmed cell death ligand 1 (PD-L1), which have been linked to defects in the antigen-presenting capacity of dendritic cells and a state of immune tolerance, respectively [23,24]. In addition, the bone marrow microenvironment in multiple myeloma has been shown to be immunosuppressive, providing a protective niche for the proliferation, migration, survival, and acquisition of drug resistance by malignant plasma cells [25,26,27,28,29]. Previous studies revealed that secreted inflammatory cytokines support the growth of immunosuppressive cells such as myeloid derived suppressor cells (MDSCs), tumor-associated macrophages (TAMs), and regulatory T-cells (Treg). Bone marrow stromal cells (BMSCs), osteoclasts (OCs), and plasmacytoid dendritic cells (pDC), as well as cytokines, i.e., interleukin-6 (IL-6), Macrophage colony-stimulating factor (M-CSF), interleukin-10 (IL-10), tumor necrosis factor beta (TGFβ), C-C Motif Chemokine Ligand 2 (CCL2), and vascular endothelial growth factor (VEGF), also play important roles in maintaining an immunosuppressive environment in the bone marrow of multiple myeloma patients [25,27]. These findings suggest that an effective anti-myeloma treatment will require not only targeting the malignant plasma cell itself but also restoring the anti-tumor responses of immune effector cells via blockade of tumor evasion and disruption of inhibitory signals on effector cells.

Monoclonal antibody-based treatments which provide additional effector cell-mediated tumor killing mechanisms when compared with targeted small molecules are successful therapeutic strategies for cancer. Monoclonal antibodies targeting specific surface antigens on cancer cells can kill the targeted cell via various effector-dependent and -independent mechanisms. Thus far, therapeutic IgG1-based monoclonal antibodies are designed to induce effector-mediated tumor cell lysis, including antibody-dependent cellular cytotoxicity (ADCC), complement-dependent cytotoxicity (CDC), and/or antibody-dependent phagocytosis (ADPC). Dependent on target antigens, therapeutic antibodies also act via receptor blockade to inhibit cell growth, induce apoptosis, or specifically deliver drug, radiation, or cytotoxic agent. Furthermore, the Fc region of antibodies plays an important role in mediating the killing of cancer cells via activation of certain immune cells (NK cells or cytotoxic T cells) as well as the induction of phagocytosis, CDC or ADCC [30,31]. For the treatment of hematological malignancies, the development of the anti-CD20 monoclonal antibody rituximab represents an important landmark and opened new venues for targeted cancer immunotherapies. Due to the success of rituximab in the treatment of B-cell lymphomas, the search for novel monoclonal antibodies for myeloma treatment has been rigorously pursued. Only small percentages of MM patients express CD20, so rituximab is not generally useful in myeloma [32,33]. Following the demonstration of promising preclinical and clinical activities [34,35,36], two monoclonal antibodies targeting CD38 (daratumumab) [37] and SLAMF7 (elotuzumab) [38] were approved by the Food and Drug Administration (FDA) to treat patients with relapsed and refractory multiple myeloma in late 2015.

**Figure 1:**
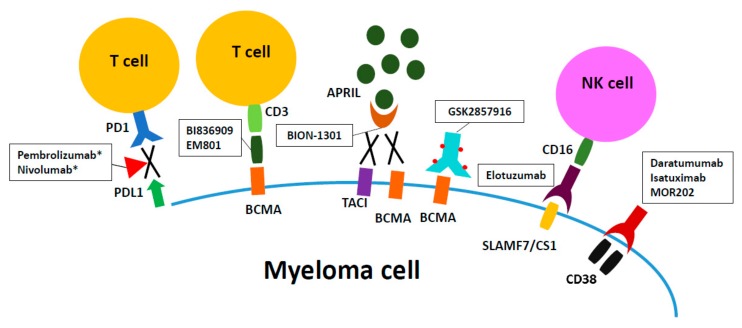
Therapeutic monoclonal antibodies for current multiple myeloma treatment. Daratumumab and elotuzumab, targeting CD38 and signaling lymphocytic activation molecule F7 (SLAMF7)/CS1, respectively, are naked IgG1 monoclonal antibodies which have been approved by FDA for treatment of relapsed/refractory multiple myeloma in late 2015. GSK2857916 is an antibody-drug conjugate composed of Fc-engineered IgG and a potent anti-tubulin drug MMAF. EM801 and BI836909 are bispecific T cell engagers targeting B-cell maturation antigen (BCMA) on myeloma cells and re-directing CD3+T cells to kill myeloma cells. BION-1301 exerts anti-myeloma activity by blocking the binding of APRIL to its cognate receptors BMCA and Transmembrane activator and CAML interactor (TACI), thereby abrogating growth, survival, and immuno-suppression signaling for myeloma cells. Clinical investigations of above agents are ongoing. * The clinical trials of PD-1 inhibitors (pembrolizumab and nivolumab) have been hold by Food and Drug Administration (FDA).

Here, we focus on monoclonal antibodies showing multiple anti-myeloma mechanisms, including those with immunomodulatory effects (Figure 1). The preclinical and clinical data of these monoclonal antibodies are summarized. We also discuss other molecular targets with therapeutic potential in multiple myeloma.

## 2. Targets and Monoclonal Antibodies

### 2.1. CD38

CD38 is a 46-kDa type II transmembrane glycoprotein with a short N-terminal cytoplasmic tail (20-aa) and a long extracellular domain (256-aa), which is identified on the surface of several cells of the immune system [39]. The intensity of CD38 expression is increased when lymphocytes are activated, and its expression is found in the majorities of hematopoietic linage cells. It is widely represented on lymphoid and myeloid cells, but absent from most mature resting lymphocytes. It catalyzes production of secondary messengers that affect Ca^2+^ mobilization. The biological function of CD38 has been linked to the regulation of calcium homeostasis in CD38-expressing lymphocytes [40]. Moreover, CD38 plays multiple but independent biologic roles since it acts both as a bifunctional enzyme responsible for the synthesis and hydrolysis of cyclic ADP-ribose, and as a signal-transducing surface receptor. CD38-/- mice are viable, without histological or pathological abnormalities. The natural ligands for CD38 are nicotinamide adenine dinucleotide (NAD)+, the substrate for its ecto-enzyme activity (ADP-ribosyl cyclase), and CD31/PECAM. Binding of CD31/PECAM and CD38 induces tyrosine phosphorylation and downstream signaling events regulating proliferation and cytokine release in lymphocytes. Regarding CD38 and multiple myeloma, previous studies have revealed that this glycoprotein is strongly and homogeneously expressed in terminally differentiated normal and malignant plasma cells [41,42]. A role of CD38 in the pathophysiology is postulated due to its high expression on a variety of hematological malignancies [43] including multiple myeloma [42,44], B- and T- acute lymphoblastic leukemia (ALL) [45,46], Non-Hodgkin lymphoma (NHL) [47], Acute myeloid leukemia (AML) [48] and Chronic Lymphocytic Leukemia (CLL) [49,50]. A recent study also suggested that CD38 enzymatic activity may be associated with immunosuppression in patients with multiple myeloma patients, due to its involvement in the production of immunosuppressive adenosine (ADO) [51].

#### 2.1.1. Daratumumab

Daratumumab, a human IgG1-kappa monoclonal antibody, was the first naked CD38 monoclonal antibody to be further developed for clinical use following demonstration of promising anti-myeloma activity in preclinical studies with cell lines and animal models. In preclinical studies, it was found that daratumumab (formerly named HumaxCD38) kills CD38-expressing lymphoma and myeloma cells by various mechanisms including CDC, ADCC, ADCP, and induction of apoptosis after Fcγ receptor-mediated crosslinking with anti-human IgG1 secondary antibody [36,52,53]. In other CD38-expressing cells such as human NK cells, B and T cells, activated T cells or monocytes, the process of CDC cytotoxicity induced by daratumumab was not seen [36]. CD38 expression is relatively low on the cell membrane of these cells when compared with malignant plasma cells from multiple myeloma patients. Possible explanations for this therapeutic index include increased expression of complement regulatory proteins on the surface membrane of these cells or the need for a minimum threshold level of antigen expression needed to activate CDC [54,55]. Unlike CDC, daratumumab-mediated ADCC was seen in these cells and in primary tumor cells. In another study, ADCC was significantly enhanced if mononuclear effector cells derived from healthy peripheral blood donors were pretreated with lenalidomide, associated with activation of NK effector cells by lenalidomide [56]. Daratumumab also demonstrated potent antitumor activity in CD38-expressing xenografts in immune deficient mice [36], suggesting that daratumumab may mediate non-immune mediated anti-tumor activities in vivo.

A recent correlative study analyzed via flow cytometry on bone marrow and peripheral blood samples from participants in a clinical trial showed that daratumumab treatment rapidly depleted CD38 high-expressing immunosuppressive regulatory T cells (Treg) and B cells (Breg), as well as myeloid-derived suppressor cells (MDSC) [57]. In contrast, the numbers of immune effector cells such as helper and cytotoxic T cells increased [57,58]. CD38 levels are heterogenous among sub-populations of hematopoietic lineage cells. It is found to be expressed at significantly higher levels in Treg, Breg, and MDSCs, when compared with normal T, B, NK, and monocytes. This study therefore indicates that daratumumab quickly reduces these key immune inhibitory cellular components, thereby relieving their suppressive immune function and increasing effector cell-induced tumor cell lysis. Thus, in addition to its multiple FcR-dependent tumor cell killing mechanisms, daratumumab further blocks immunosuppressive cellular components, which may provide for more long-term responses [57].

##### Clinical Trials of Daratumumab

In phase 1–2 study, daratumumab monotherapy was administered to heavily pretreated patients with relapsed or refractory multiple myeloma (with a median of 5.5 lines of prior therapy, 75% refractory to lenalidomide and bortezomib) [37]. There were 32 patients enrolled in the dose-escalation phase. Daratumumab was administered weekly for 8 weeks, at doses ranging from 0.005 to 24 mg/kg. The maximum tolerated dose was not reached. In the expansion phase, 72 patients received 8 mg/kg or 16 mg/kg of daratumumab. The patients who received 16 mg/kg of daratumumab showed a better overall response rate (36% vs. 10%) and longer median progression-free survival (5.6 vs. 2.4 months) than patients treated with 8 mg/kg. Infusion-related reactions were the most frequently reported adverse effects, occurring in 71% of patients in the dose expansion group, mostly grade 1 or 2. In terms of hematologic adverse effects, neutropenia was most common, occurring in 12% of patients in the 16 mg/kg cohort [37]. Results of this small clinical study demonstrated the impressive activity of monotherapy with this agent in patients with no other available treatment options, leading the approval by the FDA in 2015 [37].

In the phase 2 SIRIUS study, 106 patients with multiple myeloma refractory to proteasome inhibitors and IMiDs (with a median of 5 lines of prior treatment) received daratumumab at 16 mg/kg. The overall response rate was 29.2% [59]. The time to response and median progression-free survival were 1.0 month and 3.7 months, respectively. The 12-month overall survival was 64.8%, and the median overall survival was 17.5 months. Infusion-related reactions were noted in 42% of patients, mostly grade 1 or 2. The most common grade 3 or 4 adverse effects were anemia (24%), thrombocytopenia (19%) and neutropenia (12%) [59].

With respect to combination studies, the results of two phase 3 studies have been published [60,61]. In the CASTOR trial, 498 patients with relapsed or refractory multiple myeloma (≥1 prior line of therapy) received bortezomib plus dexamethasone with or without daratumumab [60]. The incorporation of daratumumab in the treatment regimen significantly improved the overall response rate (82.9% vs. 63.2%, *p* < 0.001), the 12-month progression-free survival (60.7% vs. 26.9%), and the median progression-free survival (not reached vs. 7.2 months, *p* < 0.001). The most common grade 3 or 4 adverse events reported in the daratumumab group were thrombocytopenia (45.3%), anemia (14.4%), and neutropenia (12.8%). Infusion related reactions were noted in 45.3% of patients from the daratumumab group.

In another phase 3 trial, the POLLUX study, daratumumab proved to be a good therapeutic combination with lenalidomide and dexamethasone [61]. In this study, 569 patients who had received one or more lines of anti-myeloma treatment received lenalidomide with or without daratumumab. Adding daratumumab to lenalidomide and dexamethasone was associated with better response rates (93% vs. 76%, *p* < 0.0001), complete response rates (43.1% vs. 19.2%, *p* < 0.0001) and progression-free survival at 12 months (83.2% vs. 60.1%). The daratumumab group also showed a higher rate of minimal residual disease negativity (22.4% vs. 4.6%, *p* < 0.001). The most common grade 3 or 4 adverse effects in the daratumumab group were neutropenia (51.9%), thrombocytopenia (12.7%) and anemia (12.4%). Infusion-related reactions were noted in 47.7% of patients of the daratumumab group [61].

An important finding from both CASTOR and POLLUX was that the benefit of the addition of daratumumab to existing doublets persisted regardless of the number of prior lines of therapy. Greater benefit was seen when the triplet modality was used earlier in the disease course. Although close to half of the patients experienced daratumumab-related infusion reactions, >90% of these events occurred only upon the first infusion. This observation indicated that repeated dosing is safe. Both regimens were approved in November 2016 by the FDA for the treatment of multiple myeloma patients who have received at least one prior therapy. In addition, the unprecedented results stimulated studies for the detection of minimal residual disease (MRD) with next generation sequencing (NSG) and next generation flow-cytometry. The new MRD categories are currently being standardized to report across clinical trials in order to validate their importance as key prognostic markers and to guide treatment decisions.

#### 2.1.2. Isatuximab (SAR650984)

Isatuximab, formerly called SAR650984 [62], is a novel humanized IgG1-kappa anti-CD38 monoclonal antibody currently under clinical development. Isatuximab was selected because of its direct induction of apoptosis in CD38-expressing lymphoma cell lines, in addition to its multiple effector cell-dependent cytotoxicity. In a preclinical study, isatuximab induced cell death in myeloma cell lines by ADCC, CDC, and ADCP, as well as the induction of tumor cell death in a CD38-dependent manner [62]. It is the latter activity which differentiates isatuximab from other therapeutic CD38 monoclonal antibodies because tumor cell death is directly induced by isatuximab in the absence of immune effector cells. It has similar half maximal effective concentrations (EC50 ~ 0.1 μg/mL) and maximal binding as daratumumab but MOR03087 (MOR202) (discussed later in this article) has a lower apparent affinity (EC50 ~ 0.3 μg/mL) [63]. These three CD38 monocloncal antibodies were equally potent at inducing ADCC against CD38-expressing tumor cells [63]. Daratumumab demonstrated superior induction of CDC in Daudi lymphoma cells as determined by flow cytometry, when compared with other CD38 antibodies in current clinical development. Specifically, isatuximab, more potently than daratumumab, inhibits ecto-enzyme function of CD38. It produced the largest inhibition of cyclic GDP-ribose (cGDPR) production, indicating a higher modulation of CD38 cyclase activity.

In in vivo studies using the same multiple myeloma cell lines xenografted in Severe combined immunodeficiency (SCID) mice, isatuximab showed more potent anti-myeloma activity than bortezomib [62]. Importantly, without the addition of Fc crosslinking agents or effector cells, isatuximab induced homotypic aggregation-associated multiple myeloma cell killing in a CD38-dependent manner [64]. In contrast, under similar conditions in ex vivo co-cultures, daratumumab shows no direct toxicity against multiple myeloma cells. Significantly, its F(ab)’2 fragments, just like the full-length version of isatuximab, could trigger lysosome-dependent cell death via upregulation of lysosome related protease cathepsin B and the translocation of lysosomal-associated membrane protein 1 (LAMP1) from lysosome to cell membrane, as well as increased reactive oxygen species. This effect was preferentially seen in myeloma cells expressing elevated levels of CD38 regardless of p53 mutation, which represents a key feature of most resistant patient group. Isatuximab specifically induced lysosome-dependent cell death by enlarging lysosomes and increasing lysosomal membrane permeability, despite the presence of protective IL-6 or bone marrow stromal cells. Furthermore, the addition of pomalidomide augments the direct and indirect killing effects of isatuximab in pomalidomide/lenalidomide-resistant myeloma cells [64]. Caspase 3/7-mediated apoptosis in drug-resistant myeloma cells was synergistically enhanced when both Isatuximab and pomalidomide were added together. Pomalidomide also increased ADCC mediated by isatuximab, further supporting ongoing phase III combination clinical trials.

Most recently, the effects of isatuximab on immune cell populations in the bone marrow microenvironment were investigated, since CD38 is widely expressed on hematopoietic cells [65]. First, significantly increased levels of CD38 were shown on the cell membrane of Tregs (CD4^+^CD25^high^Foxp3^+^) and myeloma cells when compared with conventional T (Tcon, CD4^+^CD25^−^) cells containing the majority of effector T cells. Higher CD38 expression and higher CD38^+^ subsets were identified on the cell membrane of Tregs (CD4^+^CD25^high^Foxp3^+^) versus Tcon, in accord with findings from a recent study of daratumumab trials [57]. Importantly, following isatuximab treatment, the percentage and function of CD38 high-expressing Tregs were decreased via the induction of apoptosis and decreased proliferation. In parallel, isatuximab blocked Treg-inhibited growth of conventional T cells in a dose-dependent manner. Longer ex vivo cocultures in the presence of low dose lenalidomide or pomalidomide further increased CD38 levels and percentages of the CD38^+^ sub-population in the viable Tregs. This result indicates that IMiDs can enhance the sensitivity of this immune inhibitory subset to isatuximab, providing an additional mechanism to support combination trials of these reagents to sustain T effector cell function. Significantly, isatuximab augmented degranulation of NK and CD8+ T effector cells leading to increased multiple myeloma cell lysis, which was further enhanced by pomalidomide or lenalidomide. Isatuximab also reduced Foxp3 and IL10, associated with the inhibitory function of Tregs, and restored the proliferation of CD4^+^CD25^−^ naive T cells. Importantly, patients with multiple myeloma have elevated CD38high Tregs that block the proliferation of Tcons; and multiple myeloma cells convert Tcon into Tregs in ex vivo co-cultures. Myeloma cells induce the generation of Tregs (iTreg) which also highly express CD38 in addition to other known Treg markers. This study further showed that Tregs can be induced by cell-to-cell contact-dependent and -independent interactions between myeloma cells and Tcons, mimicking increased frequency of Tregs in MM patients versus normal donors. These iTregs generated in ex vivo co-cultures show significantly elevated CD38 and Foxp3 levels when compared with Tcons. They still demonstrate inhibitory function and significantly decrease proliferation of Tcons, which is overcome by isatuximab [65]. In a similar fashion as occurred in multiple myeloma cells overexpressing CD38, isatuximab preferentially targets CD38high-expressing Tregs of patient MM cells [65]. These results are in accord with a recent report showing that these immune inhibitory CD38high subsets were rapidly depleted by daratumumab in a recent correlative study [57]. These studies indicate that isatuximab can mitigate the immunosuppressive bone marrow microenvironment, thereby further restoring anti-multiple myeloma immunity.

Clinical Trials of Isatuximab

In a phase 1b dose-escalation study, isatuximab was administered in combination with lenalidomide and dexamethasone to treat patients with relapsed and refractory multiple myeloma (with a median of five prior regimens) [66]. Isatuximab was given at different doses and in two schedules (3, 5 or 10 mg/kg every 2 weeks (Q2W) or 10 or 20 mg/kg weekly for 4 weeks, then Q2W thereafter (QW/Q2W)). The maximum tolerated dose was not reached in this study. The analysis of efficacy revealed that the overall response rate was 56%. The median progression-free survival was 8.5 months. The most common treatment-related adverse events were fatigue and nausea, mostly grade 1 or 2. Infusion related reactions were noted in 56% of patients, mostly grade 1 or 2, and mainly occurred during the first infusion [66].

The combination of isatuximab and pomalidomide/dexamethasone is also generally well tolerated and clinically active in patients with heavily pre-treated relapsed and refractory multiple myeloma [67]. Of the eight patients who achieved at least PR up to 10 mg/kg, all continued to respond without confirmed disease progression at data cut-off. The pharmacokinetic (PK) parameters of isatuximab were not affected by co-administration with pomalidomide/dexamethasone. Data are continuing to be collected for longer-term follow up, including the 20 mg/kg cohort. A Phase III trial to evaluate isatuximab plus pomalidomide/dexamethasone is planned.

#### 2.1.3. MOR03087 (MOR202)

MOR202 (HuCAL) is a novel fully human anti-CD38 IgG1 monoclonal antibody. In preclinical studies, MOR202 killed CD38-expressing cell lines and primary myeloma cells from patients by ADCC and ADCP. In SCID-mouse xenograft models, MOR202 inhibited tumor growth [68]. Furthermore, the addition of lenalidomide to MOR202 activated immune effector cells and augmented ADCP-mediated cytotoxicity [69]. Pomalidomide also enhances the cytotoxicity of MOR on MM cells [70].

##### Clinical trials of MOR03087 (MOR202)

In a phase I/II dose-escalation clinical trial, MOR202 was administered alone or combined with IMiDs (lenalidomide or pomalidomide) to treat 66 patients with relapsed or refractory multiple myeloma (more than 2 lines of prior therapy) [71]. MOR202 was infused for 2 h. The maximum tolerated dose was not reached in this study. Of 16 evaluable patients in the MOR202 monotherapy cohort, 3 patients (19%) showed partial responses and 2 patients (13%) showed very good partial responses. In the MOR202/lenalidomide cohort, 5 of 7 patients exhibited partial responses. In the MOR202/pomalidomide cohort, 3 of 5 patients showed a response to treatment, including 2 complete responses. Infusion-related reactions were seen in 3 patients (3/31, 10%), all occurring during the first infusion and less than grade 2.

#### 2.1.4. Side Effects of CD38 Monoclonal Antibody

Some important clinical scenarios should be considered when using daratumumab to treat myeloma patients. First, daratumamab can be detected as an individual monoclonal band, which would interfere with serum immunofixation electrophoresis tests (IFE) [72,73]. To address this issue, daratumumab IFE reflex assay was developed to abrogate interference. Second, daratumumab has been shown to interfere with evaluation of bone marrow aspirates by multiparameter flow cytometry in a small study [74]. There were no CD38 or CD138 events detected in two patients treated with daratumumab. This was contrasting to the finding of the aspirate morphology and immunohistochemical study, which showed abnormal plasma cells positive for CD38 or CD138. Utilization of antibodies binding to different epitopes of CD38 may address this issue. Third, daratumumab also interferes with blood typing because CD38 is expressed on human red blood cells. Daratumumab binds to CD38 antigen on the reagent blood cells and leads to a positive indirect Coombs test. Treatment of dithiothreitol on reagent red cells with to denatures CD38 on the surface represents a potential method to circumvent this issue.

To prevent an infusion related reaction, medications such as glucocorticoid, antihistamine and acetaminophen can be administered before infusion during the initial cycles of treatment [75,76,77]. If an infusion related reaction occurs despite the administration of premedication, the infusion should be held until the appropriate symptom management is done and symptoms have resolved. After the resolution of symptoms, daratumumab can be restarted at a lower infusion rate.

### 2.2. SLAMF7/CS1

SLAMF7 (signaling lymphocytic activation molecule F7), previously known as CS1 (cell surface 1), is a cell surface glycoprotein that is a member of the signaling lymphocytic activation molecule family [78]. SLAMF7 is expressed on NK cells, activated monocytes, T cell subsets, and normal plasma cells. The biological function of SLAMF has been linked to the regulation and activation of NK cells [78,79]. Importantly, >90% of patients with myeloma cells expressed SLAMF7 messenger RNA (mRNA) and protein, regardless of disease status and treatments [34,35]. High SLAMF7 protein expression on the cell surface of multiple myeloma cell lines and patient myeloma cells spurred the development of monoclonal antibodies targeting SLAMF7 for the treatment of this cancer.

#### 2.2.1. Elotuzumab

Elotuzumab is a humanized IgG1-kappa monoclonal antibody that targets SLAMF7. In a preclinical study, elotuzumab specifically bound to CD138^+^ myeloma cells, natural killer (NK), NK-like T cells, and CD8^+^ T cells, but not to hematopoietic CD34^+^ stem cells. The major mechanism for the anti-myeloma activity of elotuzumab was the induction of dose-dependent NK cell-mediated ADCC [34,35], since elotuzumab did not induce CDC against myeloma cells. In a mouse xenograft model, elotuzumab showed in vivo efficacy mainly via NK effector cell-mediated toxicity [34]. In addition, it inhibited the adhesion of myeloma cells to bone marrow stromal cells, thereby blocking their proliferation and survival [34]. The anti-myeloma ADCC effect of elotuzumab was further enhanced by pretreatment with lenalidomide or bortezomib [34,80], providing the rationale for the combination trial of elotuzumab with lenalidomide/dexamethasone [38] or bortezomib [81].

##### Clinical Trials of Elotuzumab

In a phase I dose-escalation study, elotuzumab monotherapy was administered to thirty-five patients with relapsed or refractory myeloma (≥2 prior therapies, median 4.5) [82]. Elotuzumab was administered in doses ranging from 0.5 to 20 mg/kg once every two weeks. The maximum tolerated dose was not reached. The treatment response, evaluated according to the European Group for Blood and Marrow Transplantation myeloma response criteria, showed that nine patients (26.5%) had stable disease. Common adverse events such as chills, fever and flushing were generally mild to moderate in severity (grade 1 or 2). Before the protocol amendment, 13 of 25 treated patients suffered from infusion-related reactions. This study found that CS1 on myeloma cells was highly saturated (>95%) with antibodies at dose levels of 10 and 20 mg/kg without dose-limiting toxicity. Two more two phases 1 studies were conducted to evaluate the efficacy of combining elotuzumab with other anti-myeloma agents [81,83]. In the first study, patients with relapsed and refractory multiple myeloma (≥1 prior therapies (median 3), pretreated with lenalidomide were eligible) were treated with elotuzumab, lenalidomide and dexamethasone [83]. The overall response rate was 82%, with 29% showing at least a very good partial response (VGPR) [83]. In the second study, elotuzumab was combined with bortezomib to treat patients with relapsed and refractory multiple myeloma (1 to 3 prior treatments (median 2), pretreated with bortezomib were eligible) [81]. The overall response rate was 48%, and the median time to progression was 9.46 months.

In the pivotal phase III ELOQUENT-2 study, 646 patients with relapsed or refractory multiple myeloma (1 to 3 prior lines of treatment) received lenalidomide plus dexamethasone with or without elotuzumab [38]. Patients with prior lenalidomide treatment were enrolled if the best response seen was a partial response or better. The elotuzumab group showed a higher overall response rate than the control group (79% vs. 66%, *p* < 0.001). After a median follow-up of 24.5 months, the elotuzumab group showed better progression-free survival when compared with the control group (19.4 vs. 14.9 months, *p* < 0.001). Infection was noted in 81% and 74% of patients in the elotuzumab and control groups, respectively. The most common grade 3 or 4 adverse effects in the elotuzumab group were lymphocytopenia, neutropenia, fatigue, and pneumonia. With steroid premedication, infusion-related reactions occurred in 10% of patients of the elotuzumab group, mostly grade 1 or 2 [38].

### 2.3. PD-1/PD-L1

The immune checkpoint inhibitor programmed cell death protein 1 (PD-1)/programmed cell death ligand 1 (PD-L1) pathway plays a significant role in the evasion of host immunity by tumor cells [84]. PD-1 is a cell surface receptor of the immunoglobulin superfamily and is expressed on T cells, B cells, and NK cells [84,85]. Blockade of the PD-1 pathway may restore the cytotoxic function of T cells against myeloma cells in vitro [86]. Furthermore, T cells produce INFγ, which upregulates PD-L1 expression on tumor and infiltrating immune cells, forming a feedback loop that generates a PD-1 signal maintaining immunosuppression [87]. PD-L1 is highly expressed on malignant plasma cells from myeloma patients, especially in those with relapsed and refractory disease, but not on normal plasma cells [86,88]. In multiple myeloma, the expression of PD-L1 can be induced by IL-6, and higher levels of PD-L1 expression are associated with increased anti-apoptotic ability and more aggressive behavior of myeloma cells. The binding of PD-1 to PD-L1 has been associated with drug resistance by myeloma cells [89]. Significantly, PD-L1 is also expressed on cells with immunosuppressive effects that support the growth of myeloma cells in the bone marrow microenvironment, such as pDCs and MDSCs [86,90,91], leading to T cell anergy upon cellular contact. PD-L1 is further induced on multiple myeloma cells by a proliferation-inducing ligand (APRIL) [28,92] or the contact with bone marrow accessory cells [65,91,93]. It is also expressed on osteoclasts which support myeloma cell growth and survival in addition to the induction of bone lesions [28]. In preclinical studies, blockade of the PD-1/PD-L1 pathway was shown to inhibit myeloma cell growth mediated by bone marrow stromal cells [93]. These studies suggest that targeting PD-1/PD-L1 is promising immunotherapeutic strategy to overcome the ability of tumor cells to evade host immunity.

Since lenalidomide and pomalidomide (IMiDs) represent an efficient clinical approach in MM treatment to improve patients’ survival, studies on the regulation of PD-1/PD-L1 pathway by IMiDs have been reported [93,94]. In addition to promoting tumor apoptosis, IMiDs activate T and NK cells, thereby increasing NK-mediated tumor recognition and killing. IMiDs stimulate T cell proliferation and cytokine secretion, decrease the expression of PD-1 on both T and NK cells in MM patients, as well as decrease both PD-1 and PD-L1 on MM cells. This leads to the inhibition of the negative signal induced by PD-1/PD-L1 axis on NK and T cells, restoring NK and T cell cytotoxic functions [91]. Thus, the combination of IMiDs with anti-PD-1/PD-L1 blocking strategies could represent a promising approach to re-establish the recognition of myeloma cells by exhausted NK and T cells to induce effective immune response. A recent study showed that PD-1/PD-L1 blockade induces anti-multiple myeloma immune response that can be enhanced by lenalidomide, which provides the framework for clinical evaluation of combination therapy [93]. Currently, there are at least two PD-1 inhibitors, nivolumab (IgG4-kappa) and pembrolizumab (IgG4-kappa) approved for solid cancer treatment.

#### Clinical trials of PD-1/PD-L1 Inhibitors

In a phase 1b study, a PD-1 inhibitor, nivolumab, was administered to patients with relapsed or refractory hematologic malignancies, including twenty-seven patients with multiple myeloma [95]. No significant disease regression was observed. Stable disease was noted in seventeen patients, with a median duration of 11.4 weeks.

With respect to the other PD-1 inhibitor, there are two trials showing promising results. In a phase I trial (Keynote-023), pembrolizumab was combined with lenalidomide and dexamethasone to treat relapsed or refractory myeloma patients (≥2 lines of prior therapy) [96]. The maximum tolerated dose for pembrolizumab was a fixed dose of 200 mg with 25 mg of lenalidomide and low-dose (40 mg) dexamethasone. Thrombocytopenia (47%), neutropenia (41%), and fatigue (29%) were the most common treatment-related adverse effects. With a median follow-up of 287 days, 13 out of 17 patients (76%) responded to treatment, with 4 showing a very good partial response and 9 showing a partial response. The median duration of response was 9.7 months.

In a phase II study, pembrolizumab was administered with pomalidomide and dexamethasone to treat forty-eight patients with relapsed or refractory multiple myeloma (≥2 prior therapies) [97]. Autoimmune pneumonitis and hypothyroidism were seen in 13% and 10% of patients, respectively, mostly less than grade 2. The overall response rate was 60%. With a median follow-up of 15.6 months, the progression-free survival was 17.4 months. The median overall survival was not reached. A higher level of expression of PD-1 in bone marrow samples may correlate with improved progression-free survival [97]. However, two phase III trials comparing lenalidomide or pomalidomide with or without pembrolizumab have been put on hold due to excess deaths in the pembrolizumab cohort (http://www.ascopost.com/News/57813) [98].

Regarding antibodies targeting PD-L1, durvalumab is being studied alone and in combination with lenalidomide (NCT02685826) in patients with newly diagnosed multiple myeloma. Durvalumab, alone and in combination with pomalidomide (NCT02616640), is being evaluated in patients with relapsed/refractory disease. Durvalumab in combination with daratumumab or in combination with pomalidomide, dexamethasone, and daratumumab (NCT02807454). The other anti-PD-L1 antibody atezolizumab is currently being tested with daratumumab in patients with refractory multiple myeloma (NCT02431208) and in patients with asymptomatic multiple myeloma (NCT02784483). However, FDA recently placed a full clinical hold of checkpoint blockade in myeloma based on risks identified in other trials for an anti-PD-1 antibody, pembrolizumab.

### 2.4. B-Cell Maturation Antigen (BCMA)

BCMA (B-cell maturation antigen), a glycoprotein and non-tyrosine kinase receptor, is selectively expressed on the surface of mature B cells or plasma cells, but not naive B cells or most memory B cells [99,100,101]. BCMA can be induced by stimulation with cytokines during the differentiation of plasma cells [102] and plays an important role in the survival of long-lived plasma cells in the bone marrow [103,104]. It is not required for B cell homeostasis, and BCMA knockout mice is not lethal [104]. Importantly, BCMA is highly expressed in all multiple myeloma patient cells [99,100,101,105] and its expression levels correlates with disease status [105,106]. BCMA was being identified as a target of donor B-cell immunity in patients with myeloma who respond to donor lymphocyte infusion (DLI) [107]. Thus, in addition to donor T cells mediated graft-versus-myeloma response following allogenic hematopoietic stem-cell transplantation, the induction of specific antibodies against cell surface BCMA may directly contribute to tumor rejection in vivo [107]. Importantly, BCMA has a more specific expression pattern when compared with the other multiple myeloma antigens CD38 and SLAMF7. Malignant plasma cells have significantly increased BCMA when compared with normal plasma cells. Other than on plasma cells, BCMA is only detected on pDCs [105,108] which can promote myeloma cell growth, survival, and drug resistance. However, BCMA expression levels are significantly lower on pDCs when compared with plasma cells from the same individual, regardless of disease status. In contrast, all other cells including monocytic DCs, normal cells, and stem cells, do not express this antigen at mRNA and protein levels. The expression of BCMA on pDCs is significantly greater in myeloma patients than in normal individuals [105], further supporting BCMA as an ideal target for myeloma treatment. Moreover, BCMA upregulation in multiple myeloma cells has been associated with higher PD-L1 expression levels in addition to key survival proteins Mcl1, Bcl2, and Bcl-xL [92]. These data suggest that BCMA may modulate the immune response against multiple myeloma in the bone marrow microenvironment.

BCMA is shed by gamma-secretase, and soluble BCMA levels in serum are elevated and correlate with disease activity in systemic lupus erythematosus [102]. Gamma-secretase releases soluble BCMA that acts as a decoy, neutralizing its cognate ligand a proliferation-inducing ligand (APRIL). Like other multiple myeloma antigens, i.e., SLAMF7, CD138, CD38, soluble BCMA was detected in the elevated levels in the serum samples of multiple myeloma when compared with normal donors [106]. It was proposed that serum BCMA levels may be a new biomarker for monitoring disease status and overall survival of MM patients. Soluble BCMA levels may also play a pathophysiological role in multiple myeloma since they could inhibit the ligand (B cell activating factor, BAFF) binding to its membrane-bound BCMA to induce signaling and stimulate normal B-cell and plasma cell development, thereby resulting in reduced polyclonal antibody levels [109].

#### Immunotherapeutically Targeting BCMA 

BCMA antibodies were developed with ligand blocking activity that could promote cytotoxicity of multiple myeloma cell lines as naked antibodies or as antibody-drug conjugates [110]. Recently, antagonistic humanized anti-BCMA antibody-drug conjugates via a noncleavable linker with auristatins (monomethyl auristatin E, MMAE or monomethyl auristatin F, MMAF) in preclinical studies demonstrated impressive in vitro and in vivo anti-multiple myeloma activity [105,111]. The BCMA antibody MMAF (GSK2857916) directly induces potent G2-M arrest, followed by apoptosis in multiple myeloma cell lines and patient cells [105]. Compared with its MMAE counterpart, GSK2857916 did not induce any bystander cytotoxicity when evaluated in co-cultures of MM cells with bone marrow stromal cells. Importantly, GSK2857916 also induces 1-log higher ADCC and ADPC against MM cells due to its afucosylation via FcR engineering, when compared with its homolog with normal FcR fragment. A phase 1 clinical study is ongoing, and preliminary data suggested a clinical activity at higher doses in patients with recurrent disease (NCT02064387) [112]. Thus far, maximal tolerated dose has not been reached. Adverse events were manageable, with ocular toxicity emerging as the most frequent reason for dose modifications.

One potential immunotherapeutic strategy is the development of T-cell bispecific antibodies, which bind simultaneously to a surface tumor cell antigen and a T-cell receptor to induce T cell-mediated killing of tumor cells harboring the target surface antigen. A BCMA/CD3 bispecific T-cell engager (BiTE^®^, Amgen, Thousand Oaks, CA, USA) antibody (BI836909), which was made based on blinatumomab (Anti-CD19/CD3 BiTE^®^ antibody, Amgen, Thousand Oaks, CA, USA), was tested in a preclinical study using BCMA on myeloma cells as a target and CD3 on T cells as the other target [113]. Following treatment with BI836909, selective lysis of BCMA-positive myeloma cells, activation and proliferation of T cells, as well as release of multiple key cytokines related to effector T cell function (IFNγ, IL-2, IL-6, TNFα, IL-10) were noted. The anti-myeloma effect of BI836909 was not significantly affected by the presence of bone marrow stromal cells, soluble BCMA and APRIL (up to 150 and 100 ng/ml, respectively). BI836909 potently induces autologous patient myeloma cell lysis regardless of disease status. Clinical trial of BI836909 (NCT02514239) is ongoing in relapsed and refractory multiple myeloma.

An IgG-based BCMA-T cell bispecific antibody (EM801) also increased CD3^+^ T cell/myeloma cell crosslinking, followed by CD4^+^/CD8^+^ T cell activation, and secretion of interferon-gamma, granzyme B, and perforin A [101]. It induced autologous T cell-mediated cell death in 34 of 43 bone marrow aspirates from patients with myeloma, including those with relapsed or refractory disease. Pharmacokinetics and pharmacodynamics indicate weekly intravenous/subcutaneous administration of EM801. Another bispecific antibody against BCMA (BiFab-BCMA) also potently and specifically redirects T cells to lyse malignant multiple myeloma cells [114]. BiFab-BCMA lysed BCMA-positive cell lines up to 20-fold more potently than a CS1-targeting bispecific antibody (BiFab-CS1) developed in an analogous fashion. In addition, the in vitro and in vivo activities of BiFab-BCMA are comparable to those of anti-BCMA chimeric antigen receptor T cell therapy (CAR-T-BCMA) [114], which has demonstrated impressive anti-myeloma activity in at least 4 recent clinical trials [115,116].

### 2.5. A Proliferation-Inducing Ligand (APRIL)

APRIL (a proliferation-inducing ligand), a member of the tumor necrosis factor family, is one of two ligands for BCMA [117,118,119]. Compared with the other ligand, BAFF, APRIL is more plasma cell-specific because it has stronger binding affinity towards receptors on plasma cells [120]. APRIL also binds to the receptor transmembrane activator, calcium modulator and cyclophilin ligand interactor (TACI), but the expression of TACI on myeloma cells is variable and lower than that of BCMA [99,121,122]. APRIL is produced by cells in the bone marrow, including myeloid-derived cells, osteoclasts, and DCs. APRIL can promote the survival of malignant plasma cells and rescue myeloma cell lines from apoptosis after IL-6 deprivation [92,121,123,124]. APRIL also promotes cell cycle progression in myeloma cells [92,125]. Based on these findings, targeting APRIL to prevent BCMA-mediated activation of myeloma cells constitutes a potential therapeutic strategy.

#### Blocking APRIL Biotherapeutics

An antagonistic anti-APRIL antibody hAPRIL01A (01A) was generated to block APRIL binding to BCMA and TACI [126]. It prevents in vitro proliferation and IgA production of APRIL-reactive B cells, and effectively impairs the chronic lymphocytic leukemia (CLL)-like phenotype of aging APRIL transgenic mice. Importantly, this antibody blocks APRIL binding to human B-cell lymphomas and prevents the survival effect induced by APRIL. Importantly, 01A inhibits APRIL- and osteoclast-induced myeloma cell proliferation and further induces apoptosis of myeloma cells in co-cultures [92]. 01A significantly blocks the growth of myeloma cells in a SCID-hu murine model, where multiple myeloma cells grow in the bone chips implanted in SCID mice. 01A augmented the cytotoxicity mediated by IMiDs and proteasome inhibitors in the co-cultures of myeloma cells with BCMA-negative bone marrow accessory cells and effector cells. Furthermore, following 01A treatment, APRIL-induced expression of genes involved in immunosuppression, i.e., PD-1, transforming growth factor beta (TGF-β), and interleukin 10 (IL-10), is decreased in multiple myeloma cells [28,92,127]. BION-1301, the clinical candidate for 01A, will be tested soon in multiple myeloma.

### 2.6. Potential Targets 

Several additional cell surface antigens with therapeutic potential have been identified [128,129,130,131]. BAFF (B-cell activating factor) is a member of the TNFα superfamily that promotes the adhesion of myeloma cells to bone marrow stromal cells via activation of the AKT/NF-κB signaling pathway [122]. High levels of BAFF have been described in patients with multiple myeloma [121,132]. In an animal model of MM, mice treated with anti-BAFF antibody had significantly lower levels of soluble human IL-6 receptor and improved survival when compared with controls [132]. Tabalumab, a humanized monoclonal antibody targeting BAFF, was evaluated in a phase I clinical trial with bortezomib [133]. FcRH5, a B-cell lineage marker broadly expressed in myeloma, was targeted as one of the T-cell bispecific antibodies to induce T cell-mediated killing of FcRH5-expressing tumor cells [131]. PD-L1 blockade further enhanced the activity of FcRH5-CD3 T-cell bispecific antibody, suggesting the possibility for combination therapy in patients with multiple myeloma. Other therapeutic targets that are also being rigorously evaluated include IL-6 [134,135,136,137], CD40 [138], CD138 [139], MUC-1 [140] and Dickkopf-1(DKK-1) [141].

## 3. Conclusions

The development of monoclonal antibodies targeting selective multiple myeloma antigens represents an important advance in the improvement of effective immunotherapies for patients with multiple myeloma. In addition to various mechanisms mediated via FcR-expressing effector cells (ADCC, CDC or ADCP), monoclonal antibodies can produce immunomodulatory effects on immune cells in the bone marrow microenvironment by decreasing the function and number of immunosuppressive cells and restoring the tumor-killing activities of immune effector cells (Table 1). Such novel immunomodulatory effects may further lead to deepened clinical responses and improved efficacy, which is exemplified by daratumumab in recent large phase 3 clinical trials. Previous clinical trials have demonstrated that monoclonal antibodies constitute an efficacious therapeutic option even for heavily pretreated patients with relapsed and refractory multiple myeloma [142]. Either as a single agent or combined with other anti-myeloma drugs as well as immune checkpoint blockade and vaccination strategies, these antibodies will further improve the prognosis significantly. Furthermore, their acceptable safety profiles make monoclonal antibodies ideal partners to combine with other anti-myeloma agents in the search for better and more durable responses in patients with all stages, especially in early disease when the immune cells are still functional. In the case of monoclonal antibodies that have already been approved for the treatment of relapsed and refractory myeloma, their possible role as frontline treatments is being rigorously investigated. Ongoing and future studies are addressing the issue of which combinations are most effective at various stages of the disease. With the continued rapid development of novel monoclonal antibodies, we can expect transformation of the treatment landscape and associated improvement in patient outcome.

## Figures and Tables

**Table 1:** Summary of monoclonal antibodies used in the treatment of myeloma.

Target	Name of the Antibody	Anti-Myeloma Mechanism	Immunomodulatory Effects
CD38	Daratumumab	CDC, ADCC, ADCP, induction of apoptosis when crosslinked, enzymatic modulation [36]	1. Deletion of CD38^+^ Tregs and Bregs [57]
			2. Expansion of CD8^+^ cytotoxic T cells and CD4^+^ helper T cells [57]
[34,35] CD38	Isatuximab	ADCC, CDC, ADCP, direct cell death via lysosome-mediated and apoptotic pathway [62]	1. Augmentation of NK and CD8^+^ T effector cell-mediated anti-tumor immune responses [65]
			2. Reduction of Foxp3 and IL10 in Tregs [65]
			3. Restoration of proliferation and function of naive T cells [65]
CD38	MOR03087	ADCC, ADCP [69,70]	Activation of immune effector cells (Combined with IMID) [69,70]
SLAMF7/CS1	Elotuzumab	ADCC [34,35]	Activation of NK cells [79,143]
PD1	Pembrolizumab	Induction of apoptosis [86]	Activation and proliferation of T cells [86]
	Nivolumab		
BCMA	BI 836909	Potent induction of apoptosis [113]	BCMA- induced T-cell activation and cytokine release [113]
	GSK2857916	ADCC, ADCP, G2-M arrest followed by apoptosis [105]	1. Improved potency and efficacy of effector cell-mediated MM cell lysis [105]
			2. G2-M growth arrest followed by apoptosis
	EM801	Induce myeloma cell death by autologous T cells [101]	Activation of CD4^+^/CD8^+^ T cells [101]
APRIL	BION-1301	Blockage of APRIL-induced growth and survival, induction of apoptosis [92]	Decreased expression of PD-1, TGF-βand IL-1 genes) [92]

CDC, complement-dependent cytotoxicity; ADCC, antibody-dependent cell-mediated cytotoxicity; ADCP, antibody-dependent cellular phagocytosis. IMID, immunomodulatory drugs; NK, natural killer cell; SLAMF, signaling lymphocytic activation molecule F7; BCMA, B-cell maturation antigen; APRIL, proliferation-inducing ligand.
